# Editorial

**Published:** 1866-06

**Authors:** 


### EDITORIAL.

We give up a large proportion of our space to the official record of the proceedings of the American Medical Association at Baltimore. This has necessitated the postponement of considerable matter which had been prepared for the present number of the *Journal*, but we feel confident that our readers all take sufficient interest in the transactions of their highest representative body to desire the earliest and fullest report that can be issued.

The meetings of the Asssociation were marked by no unusual features. There was the ordinary amount of speech making, and its quality did not differ in any perceptible degree from the oratory of previous sessions. The ultra-specialists performed their customary antics which injured no one but the performers, and were chiefly annoying through the loss of time which they caused the Association. The discussion on cholera showed the necessity for additional observation and study of the disease. A large number of interesting papers were submitted to the sections, and will serve to enrich the forthcoming volume of transactions. The festivities of the season were fully up to the standard of Esculapian propriety. Altogether, the meeting was a success.

One, however, cannot refrain from expressing a hope that the time will soon arrive when these annual sessions shall be so conducted that their influence upon the profession may be something more impressive than is now the fact. Longer sessions; more committee work; and less random debate, would add incalcuably to the weight and dignity of the proceedings of the Association. Instead of three days of social enjoyment interlarded with medical debate, the annual meeting of the association should be a true *Congress*, in which the vexed questions of the day should be deliberately and adequately discussed, that some positive result may be arrived at as the expression of the voice of the profession, respecting all matters which are brought to the notice of the body. Such a course might detract from the brilliance and eclat of the gathering; but if this be an objection, why not add to the present system of association a higher body of deliberation, bearing to the existing form the relation which subsists between the Senate of the United States and the popular branch of the government?

---

WANTED—The February and August Nos. of this Journal for 1864. Address Eds. Journal.

